# Similar proactive effect monitoring in free and forced choice action modes

**DOI:** 10.1007/s00426-022-01644-4

**Published:** 2022-02-04

**Authors:** Christina U. Pfeuffer, Andrea Kiesel, Lynn Huestegge

**Affiliations:** 1 Cognition, Action, and Sustainability Unit, Department of Psychology, Albert-Ludwigs-Universität Freiburg, Engelbergerstrasse 41, 79085 Freiburg, Germany; 2 Department of Psychology, University of Würzburg, Würzburg, Germany

## Abstract

When our actions yield predictable consequences in the environment, our eyes often already saccade towards the locations we expect these consequences to appear at. Such spontaneous anticipatory saccades occur based on bi-directional associations between action and effect formed by prior experience. That is, our eye movements are guided by expectations derived from prior learning history. Anticipatory saccades presumably reflect a proactive effect monitoring process that prepares a later comparison of expected and actual effect. Here, we examined whether anticipatory saccades emerged under forced choice conditions when only actions but not target stimuli were predictive of future effects and whether action mode (forced choice vs. free choice, i.e., stimulus-based vs. stimulus-independent choice) affected proactive effect monitoring. Participants produced predictable visual effects on the left/right side via forced choice and free choice left/right key presses. Action and visual effect were spatially compatible in one half of the experiment and spatially incompatible in the other half. Irrespective of whether effects were predicted by target stimuli in addition to participants' actions, in both action modes, we observed anticipatory saccades towards the location of future effects. Importantly, neither the frequency, nor latency or amplitude of these anticipatory saccades significantly differed between forced choice and free choice action modes. Overall, our findings suggest that proactive effect monitoring of future action consequences, as reflected in anticipatory saccades, is comparable between forced choice and free choice action modes.

## Introduction

Goal-directed actions are only possible, because our learning history allows us to predict which action will lead to which consequence. Simultaneously, this enables us to anticipate the future consequences of our actions. These anticipations of the future effects our actions will cause are also reflected in anticipatory^1^ eye movements towards the location at which an effect will occur (Pfeuffer et al., 2016; saccade-effect congruency, SEC, effect). That is, anticipatory saccades provide a unique new way to directly assess a proactive monitoring process aimed at anticipated future effects. Here, we examined whether such anticipatory saccades emerge solely on the basis of associations between actions and their effects or whether stimuli that are predictive of the future effects also influence them. Furthermore, we investigated whether the action mode (forced choice vs. free choice responses) a person responds in also affects anticipatory eye movements or, more precisely, the proactive effect monitoring process they reflect.

Ideomotor theories of goal-directed action control posit that humans select and plan the appropriate action to achieve a desired effect based on their prior learning experiences (e.g., James, 1981, Elsner & Hommel, 2001; Hommel, 2009; Hommel & Elsner, 2009; Hommel et al., 2001; Kunde, 2001; for a review, see e.g., Pfister, 2019; Shin et al., 2010). When an action contingently leads to the same effect, bi-directional action–effect associations are formed (e.g., Elsner & Hommel, 2001). Once these bi-directional action–effect associations have been formed, anticipating a desired consequence (e.g., the light turning on) activates the bi-directional action–effect association and thereby triggers the corresponding action (e.g., pressing the light switch). For instance, when participants have experienced that their left/right key presses contingently led to high-/low-pitched tones, they are subsequently faster to respond to these tones with learning-compatible actions (i.e., actions that caused the effect tone during the learning phase) rather than learning-incompatible actions (i.e., actions that caused another effect tone during the learning phase; Elsner & Hommel, 2001). In addition, they also choose learning-compatible actions more often than learning-incompatible actions.

Moreover, the influence of anticipated effects on action selection has also been illustrated by studies on response–effect (R–E) compatibility (e.g., Kunde, 2001, 2003). When actions predictably led to visual effects on the left/right side, participants were faster when action and effect were spatially R–E compatible (e.g., right key press ► effect on the right side) rather than spatially R–E incompatible (e.g., right key press ► effect on the left side). Similar R–E compatibility effects have also been observed when actions and their subsequent effects overlapped in other dimensions (e.g., intensity, see Kunde, 2001, duration, see Kunde, 2003; see Kornblum et al., 1990, for further information on dimensional overlap). R–E compatibility effects suggest that we anticipate our actions' effects prior to acting which allows for influences of future action consequences on our actions (see also e.g., Janczyk & Lerche, 2019; Shin et al., 2010).

Interestingly, action–effect anticipation can also be assessed more directly. When our actions produce predictable visual effects on the left/right side after a short delay, we anticipatorily already move our eyes towards the location at which our actions’ future effects will subsequently appear (anticipatory saccades; Pfeuffer et al., 2016; see also Herwig & Horstmann, 2011; Huestegge & Kreuzfeldt, 2012; Riechelmann et al., 2017, 2021, for additional evidence that action–effect anticipation is reflected in eye movements; see, e.g., Land & Hayhoe, 2001; Land, 2006, 2009, for anticipatory eye movements in everyday situations that might also be related to action–effect anticipation). These anticipatory saccades towards our actions’ future effects occur spontaneously and without any instruction regarding eye movements.^2^

Saccade programming is commonly thought to be faster than the planning of manual responses. Conversely, however, at least for conditions with a sufficient delay between action and effect, anticipatory saccades mostly occurred after effect-generating manual responses in an action–effect interval between manual response and corresponding effect (see Pfeuffer et al., 2016, for a detailed discussion of the temporal relations between anticipatory saccades and manual responses). Furthermore, anticipatory saccades emerged irrespective of whether there was a significant R–E compatibility effect in manual performance measures or not (Pfeuffer et al., 2016; reflecting an influence of action–effect anticipation on action selection; see e.g., Kunde, 2001). For these and further reasons, we concluded that anticipatory saccades reflected processes that were dissociable from effects of action–effect anticipation on manual action selection (Pfeuffer et al., 2016). Instead, we suggested that anticipatory saccades reflected an anticipatory preparation for evaluating whether the actual effect matched the expected effect (for further theoretical disseminations of the idea that goal-directed action control consists not only of processes related to action selection, but also of processes related to outcome evaluation/effect monitoring, see, e.g., Band et al., 2009; Chambon & Haggard, 2013; Hommel, 2015, 2017; Verschoor et al., 2013; for cybernetic comparator models of movements including control loops based on the comparison of expected and actual effects, see, e.g., Wolpert & Flanagan, 2001; Wolpert & Ghahramani, 2000). That is, anticipatory saccades, spontaneously occurring during goal-directed action control, reflect a proactive effect monitoring process which prepares a later comparison of expected and actual effect.

We showed that anticipatory saccades emerged both for forced choice targets that indicated whether participants were to press the left or right key as well as when participants freely choose their actions (Pfeuffer et al., 2016). Yet, two important questions that are relevant both from a methodological perspective (determining which research questions can be addressed using anticipatory saccades) as well as from a theoretical perspective remained unanswered.

First, the observation of anticipatory saccades under free choice conditions unequivocally demonstrated that the effects participants chose to produce led to action–effect anticipations. Yet, under forced choice conditions, the targets themselves were equally predictive of the upcoming effects, and thus, target–effect rather than action–effect associations might have been the cause of anticipatory saccades under forced choice conditions.

Here, we thus first aimed at determining whether anticipatory saccades occurring under forced choice conditions could emerge on the basis of action–effect associations alone (Experiment 1). To do so, we used repeat/switch forced choice targets that did not themselves predict the upcoming effects. We hypothesized that anticipatory saccades would nonetheless occur, indicating that action–effect associations (at least) substantially contributed to proactive effect monitoring processes under forced choice conditions.

Moreover, forced choice tasks that require a polar response decision for one or the other response option are differentiated from free choice tasks that require an arbitrary decision between multiple equal response options (e.g., Berlyne, 1957). There is a controversy regarding whether forced choice and free choice action modes differ in terms of how actions are selected on the basis of anticipated effects (for findings suggesting differences between the two action modes, see, e.g., Herwig & Horstmann, 2011; Herwig et al., 2007; Herwig & Waszak, 2009, 2012; Naefgen & Janczyk, 2018; Waszak et al., 2005; see also Ansorge, 2002; Zwosta et al., 2013, for evidence, suggesting that R–E compatibility effects depend upon the intention to produce an effect; but see e.g., Janczyk, Dambacher, et al., 2015; Janczyk, Nolden, et al., 2015; Janczyk, Pfister, et al., 2015; Janczyk et al., 2017; Janczyk, Dambacher, et al., 2015; Janczyk, Nolden, et al., 2015; Janczyk, Pfister, et al., 2015; Pfister et al., 2011; Pfister & Kunde, 2013, for contradictory findings suggesting no difference between forced and free choice). The notion that forced choice actions, in contrast to free choice actions, are stimulus-based and do not depend upon a person's intention or will was already described by Ach (1935). He argues that in stimulus-based forced choice actions, actions are not controlled by a person's will, but determined by the stimulus alone. Differentiations between free choice actions linked to intention and forced choice actions linked to stimulus-based action control inspired, for instance, imaging and electrophysiological research that suggested that different brain regions (e.g., Goldberg, 1985; Haggard, 2008; Mueller et al., 2007; Passingham, 1993; Praamstra et al., 1995; Waszak et al., 2005) and processing steps (e.g., Fleming et al., 2009; Waszak et al., 2005) might be involved in free choice, intention-based actions as compared to forced choice, stimulus-based actions (but see, e.g., Gozli, 2019, for an argument that differentiates between forced and free choice might be merely experimental artefacts). Consequently, it was suggested that only intention-based, free choice actions should be affected by the anticipation of their ensuing effects via ideomotor mechanisms and action–effect learning (e.g., Gaschler & Nattkemper, 2012; Herwig & Waszak, 2009, 2012; Herwig et al., 2007).

Yet, numerous studies have questioned whether forced choice and free choice actions are processed qualitatively differently (e.g., Hughes et al., 2011; Janczyk et al., 2017; Pfister & Kunde, 2013; Richardson et al., 2020) and whether action selection processes differ between these two action modes (for evidence that action–effect associations can be formed and retrieved under forced choice conditions, see, e.g., Janczyk, et al., 2012, 2014, 2017; Pfister & Kunde, 2013; Kühn et al., 2009; Kunde, 2001, 2003; Pfister et al., 2011; Wolfensteller & Ruge, 2011). At present, a growing number of studies support the notion that action–effect learning, action–effect anticipation, and effect-based action selection can similarly take place under forced choice conditions (see also Richardson et al., 2020, for evidence that forced choice and free choice action representations are comparable).

Interestingly, the debate regarding differences between forced choice and free choice actions has recently been taken up in the context of effect monitoring, albeit with a very indirect measure of effect monitoring processes. Specifically, Wirth et al. (2018) found that R–E incompatible effects in a first task delayed processing of a second task relative to R–E compatible effects. This was the case both in forced choice and in free choice trials. Thus, Wirth et al.'s study informs the debate on differences between forced choice and free choice action modes by suggesting that reactive effect monitoring (i.e., the process of comparing expected and actual effect when the effect is presented) occurs both under forced choice and free choice conditions. However, in Wirth et al.'s study (which was admittedly not designed to directly compare both action modes), a comparison between forced choice and free choice actions regarding effect monitoring processes was confounded with the informational value of the effects: Effects were informative regarding response accuracy in forced choice but not free choice trials. As such, effect monitoring might have been overestimated in forced choice trials and/or underestimated in free choice trials. Furthermore, Wirth et al. assessed reactive rather than proactive effect monitoring processes (i.e., processes at effect occurrence rather than in anticipation of the future effect) and did so in an indirect manner via their impact on the processing of a secondary task. Thus, both an assessment of proactive effect monitoring as well as a more direct, online measure of effect monitoring are required to draw clear conclusions regarding the impact of action mode on effect monitoring.

To provide a fair comparison between forced choice and free choice trials and to assess potential differences between the two action modes regarding proactive effect monitoring directly for the first time, we conducted Experiment 2. There, we directly compared forced and free choice targets which themselves did not predict the future effect (i.e., effect-unpredictive targets). Specifically, response repeat/switch forced choice targets and the free choice target we used preceded each effect about equally often and thus were not indicative of the future effect. Doing so, we aimed to determine whether the corresponding action modes showed differences in terms of proactive effect monitoring processes evident in anticipatory saccades, a direct, online measure of effect anticipation and proactive effect monitoring. As we previously theorized (Pfeuffer et al., 2016), effect anticipation is at the core of both action selection and proactive effect monitoring. Thus, we hypothesized that not only action selection, but also the anticipation of an action's future effect and its proactive monitoring should be comparable between forced choice and free choice action modes. To additionally assess potential influences of target–effect associations, we added effect-predictive forced choice targets (i.e., forced choice targets that 100% predictably preceded the effect of right vs. left responses). We presumed that neither action mode nor target–effect associations would affect proactive effect monitoring and, consequently, conducted corresponding Bayesian analyses in addition.

## Experiment 1

In the forced choice experiments of Pfeuffer et al. (2016), an influence of target–effect associations (as opposed to action–effect associations only) on participants' eye movements could not be ruled out. Experiment 1 tested whether anticipatory saccades towards future effects emerged in a forced choice setting even if targets did not predict upcoming effects. To address this question, we adapted the eye-tracking R–E compatibility paradigm we previously developed (Pfeuffer et al., 2016) using targets that indicated whether participants were to perform the same response as on the previous trial (repeat target) or the opposite response (switch target). Thus, Experiment 1 allowed us to substantially extend the findings of Pfeuffer et al. (2016) by assessing whether anticipatory saccades also emerged in a forced choice setting when targets were *not* predictive of the upcoming effects (i.e., no target–effect associations were possible).

### Methods

#### Participants

Sample size estimations were conducted on the basis of the mean effect size of the SEC effect (relative frequency of anticipatory saccades towards as compared to away from the future effect) reported in Pfeuffer et al., (2016; *d* = 0.79 for the one-sample t test comparing the mean of the three experiments’ SEC scores to 50%). 12 participants would suffice to find an effect of this size in the corresponding one-sample t test against 50% with α = 0.05 and 80% power (GPower; Erdfelder et al., 1996; Faul et al., 2007). We, however, assumed that the SEC effect might be reduced if target–effect associations additionally played a role. Therefore, and for comparability with Pfeuffer et al. (2016), we aimed for about 20 participants. For reasons of counterbalancing, 24 participants (17 female, 7 male, 3 left handed, 7 left eye dominant, mean age = 24.4 years, SD = 3.9) took part after providing written informed consent and were included in the analyses. All participants included in the analyses had normal or corrected-to-normal vision. For their participation, they received either course credit or 7€. The data of ten additional participants were initially collected, but these participants were replaced in the final sample. Two of these participants were replaced as the research assistant had accidentally given the wrong instruction and another four participants were replaced as they either did not finish the experiment and/or their eye movements could not be tracked reliably throughout the entire experiment. Another two additional participants were replaced due to high error rates (> 3 SDs above the sample mean). Finally, two participants reported that they had intently inhibited their eye movements to prevent measurement and were also replaced.

#### Stimuli and apparatus

Participants sat approximately 60 cm from a 24″ LCD screen (1920 pixels × 1080 pixels, 144 Hz) in a dark, sound attenuated laboratory room. Head movements were minimized using a chin rest and the index fingers of participants' left and right hand rested on two external keys placed in front of them to the left and right (key distance: 13.5 cm). The screen background was black throughout the experiment which was run via EPrime (version 2.0.10.3.5.3, Psychological Software Tools Inc., Sharpsburg, PA, USA).

Participants' eyes were tracked via an EyeLink 1000 Plus Desktop Mount (SR Research Ltd., Ontario, Canada). Corneal reflection and pupil diameter were measured via an infrared camera and eye movements (dominant eye) were sampled at 1000 Hz with a spatial resolution of 0.01° visual angle. Calibration and validation were performed before the beginning of each block.

#### Design and procedure

The experiment consisted of ten experimental blocks of 50 trials (blocks 1–5 R–E compatible and blocks 6–10 R–E incompatible or vice versa) and a preceding practice block of 36 trials (536 trials in total). Per block, participants had to respond with a right/left key press equally often. After each block, participants had the opportunity to take a self-paced break.

**Fig. 1:**
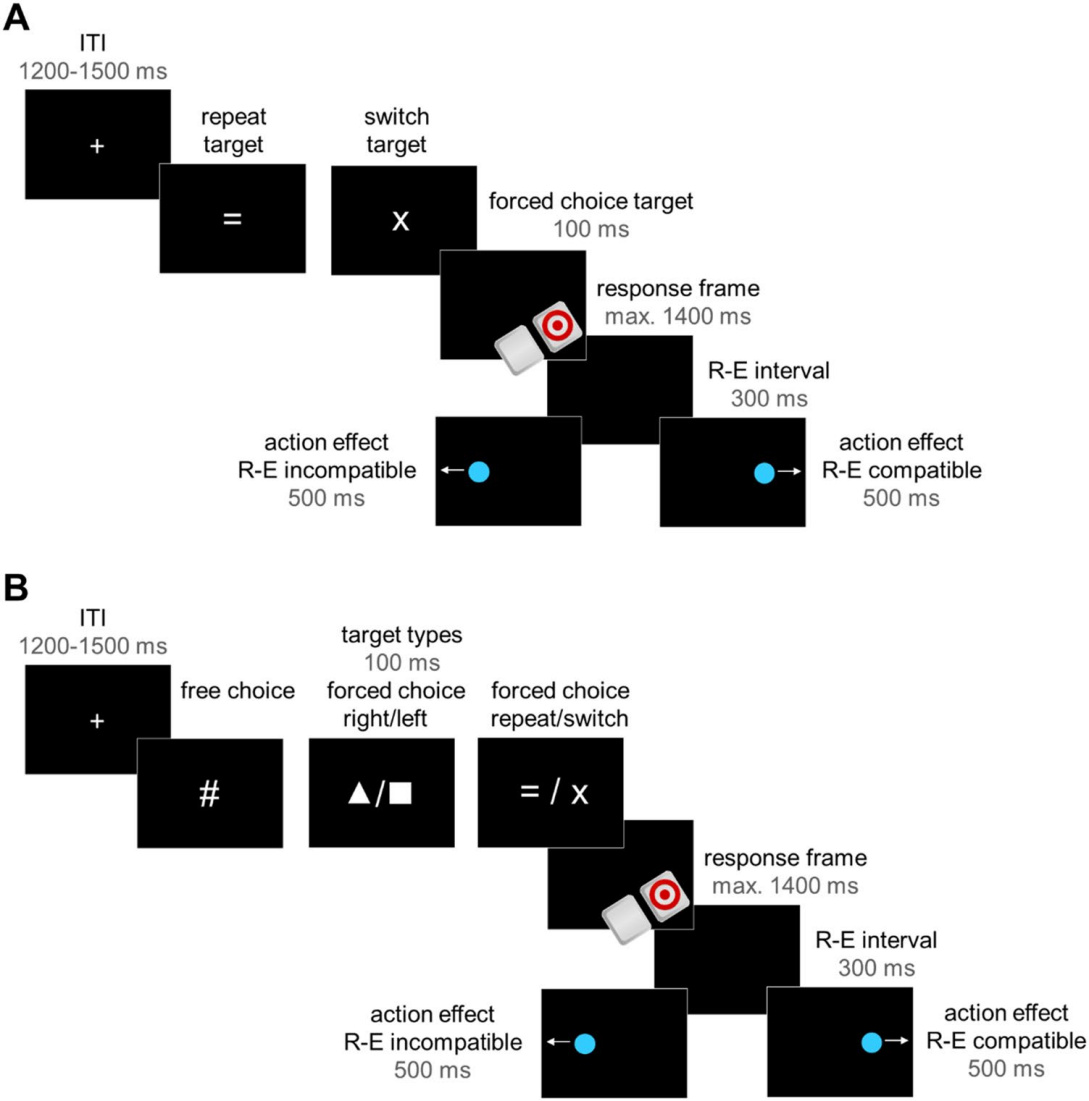
Trial structure of **A** Experiment 1 and **B** Experiment 2. Targets (Exp. 1: forced choice repeat/switch; Exp. 2: free choice vs. forced choice rigth/left vs. forced choice repeat/switch) were followed by a blank screen response frame and participants' correct left/right responses reliably triggered lateralized effects after a fixed response–effect (R–E) interval. Actions and their effects were response–effect (R–E) compatible in one half of the experiment and R–E incompatible in the other half. Trials were separated by a variable inter-trial interval (ITI)

Each trial started with an inter-trial interval (ITI) with a centrally displayed white fixation cross (0.5°; see Fig. 1A for the trial structure of Experiment 1) with a variable duration of 1200–1500 ms. The ITI was jittered to decrease the temporal predictability of the targets. Then, the target (0.7°) appeared for 100 ms. In the first trial of each block, the target was a white arrow pointing to the right or left to set an initial response. Crucially, in all subsequent trials of a block, the target was either a white “ = ” (repeat target), signaling that the same response as on the previous trials should be performed, or a white “x” (switch target), signaling that the opposite response should be performed. Thus, the reference was always the correct response on the previous trial. Participants were instructed to respond fast and accurately to the targets. Note that targets were deliberately presented for a short duration as anticipatory saccades were only assessed from target offset to effect onset (anticipatory interval, i.e., during a blank screen interval when no visual stimulation was present). After the target had disappeared, the screen went blank and participants could continue to respond to the target for another 1400 ms at maximum (response limit 1500 ms in total). As soon as participants had responded or when the time limit had passed, a blank screen response–effect (R–E) interval of 300 ms followed.

When participants had responded correctly, an effect, a blue or orange circle (1.3°, start position: 12.6° to the left/right, duration 500 ms) appeared to the right/left of the screen center and moved further towards the right/left side of the screen at a continuous pace (additional movement: 6.6°, end position: 19.1° to the left/right). One effect colour was consistently mapped to one of the responses, but the effect position associated with a response varied between the first (block 1–5) and second half (block 6–10) of the experiment. Effects appeared at spatially R–E compatible locations (e.g., left response ► effect on the left) in one half of the experiment and at spatially R–E incompatible locations (e.g., left response ► effect on the right) in the other half. That is, the position of a future effect was contingent upon the response throughout the experiment, but the R–E mapping switched after half of the blocks. R–E compatibility order and effect colour were counterbalanced across participants. During the practice block, there were no effects. Before the beginning of the experiment, participants were informed that their responses would now be followed by coloured circles, but they were informed neither about the initial spatial compatibility between their responses and the effects of these responses nor about the shift in R–E compatibility.

When participants responded prematurely (i.e., prior to target presentation), incorrectly, or not within the time limit, appropriate feedback (premature response: “zu früh!”/”too early!”, incorrect response: “Fehler!”/”error! “, response omission: “zu langsam!”/”too slow!”; printed in red) was displayed for 1000 ms instead of an effect. The trial was then aborted and the next trial ensued. At the end of each block, participants were informed about the number of premature and erroneous responses they had made as well as about the number of response omissions. They were then reminded to try and respond as fast and accurately as possible. During an initial instruction, participants were explicitly informed that the subsequent response (i.e., same vs. opposite response depending on whether the target was a repeat or switch target) had to be inferred from the correct response of the preceding trial.

Participants did not receive any information or instruction regarding their eye movements. They were only told to attend to the coloured circles they saw. Note that this might have been interpreted as an instruction to look towards the irrelevant effect once it was presented and might thus have facilitated saccades towards the effect upon effect presentation. It should, however, by no means have been interpreted as an instruction to anticipatorily saccade towards not-yet-present effects. We therefore assume that all observed eye movements during the anticipatory interval (i.e., from target offset to effect onset) occurred spontaneously and uninstructedly.

### Results

#### Data preparation and analyses

We assessed both participants' manual, effect-generating actions (reaction times, RTs, and error rates) and their eye movements (relative frequency of saccades towards future effects as well as latency and amplitude of anticipatory saccades towards the future effects). The first trial of each block, in which the repeat/switch targets could not yet be used, was excluded from all analyses. Similarly, trials with premature (< 0.1%) or omitted responses (0.3%) were excluded from all analyses. Trials with erroneous responses^3^ (5.3%) were excluded from RT and saccade analyses.

For the following Experiment 2, it was essential to also quantify the evidence in favor of a null effect in case of a non-significant effect. We therefore conducted additional Bayesian analyses. To parallel the analysis strategy of Experiment 2, we added Bayes factors ${\mathrm{BF}}_{01}$ to all non-significant results in Experiment 1 already, though less essential there, to quantify the evidence in favor of the null hypothesis. We used default prior scales using JASP for all Bayesian analyses (version 0.8.0.0, Love et al., 2015; see Rouder et al., 2009, 2017, for information on Bayesian statistics). Bayes factors $({\mathrm{BF}}_{01})$ are added to the test statistics of the respective effects. The Bayes factor ${\mathrm{BF}}_{01}$ indexes how strongly the data are in favor of the null hypothesis. Bayes factors between 1 and 3 are considered anecdotal evidence for the null hypothesis (see Jarosz & Wiley, 2014). Bayes factors between 3 and 10 are considered substantial evidence for the null hypothesis and Bayes factors between 10 and 30 are considered strong evidence in favor of the null hypothesis.

#### Manual responses

**Fig. 2:**
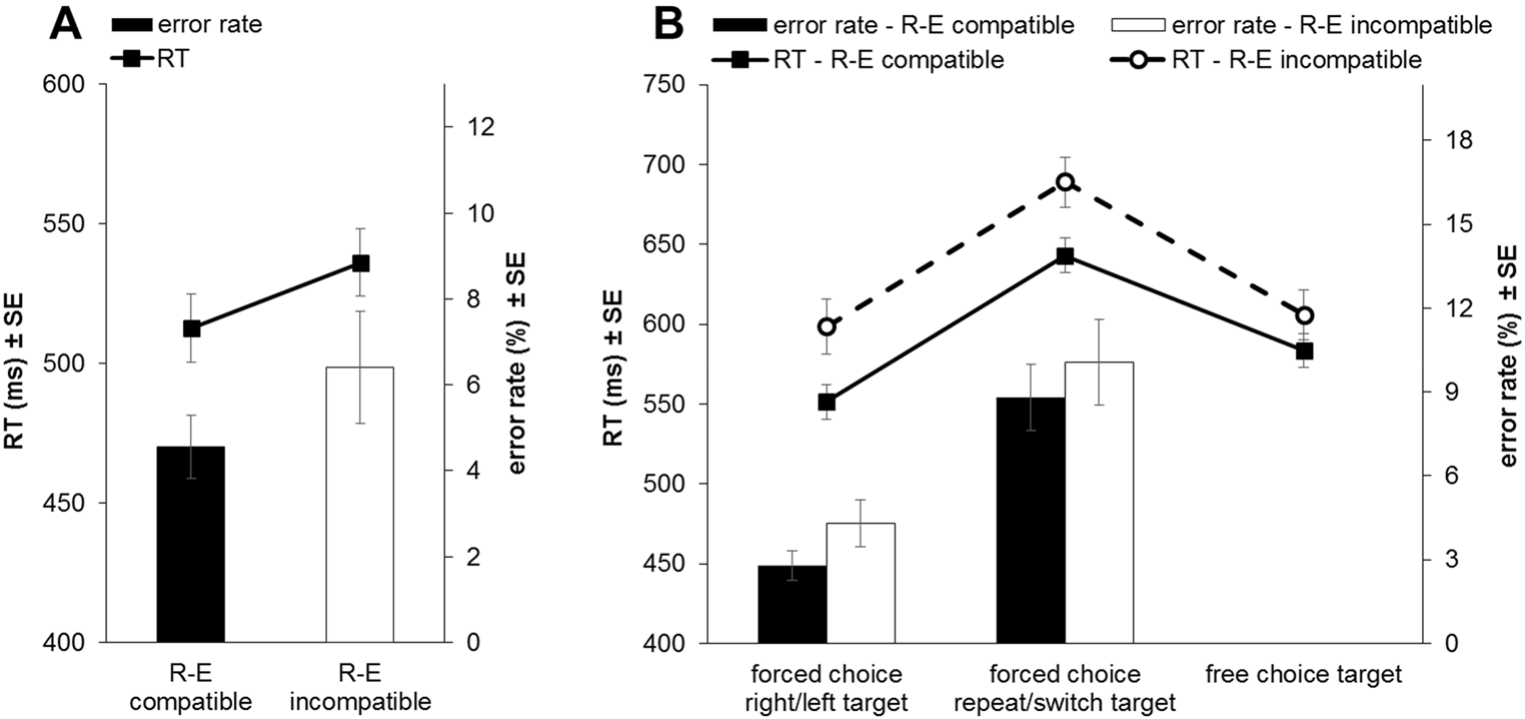
Reaction times (RTs) and error rates of manual responses in **A** Experiment 1 and **B** Experiment 2. Results are displayed separately for **A** the two R–E compatibility conditions (R–E compatible vs. R–E incompatible) and **B** the two R–E compatibility conditions and the three target types (forced choice right/left vs. forced choice repeat/switch vs. free choice). Error bars indicate the standard errors of the respective mean

For RT analysis, trials with RTs deviating by more than three standard deviations from their individual cell means were additionally excluded (1.6%). Paired t tests compared R–E compatible and R–E incompatible trials regarding reaction times and error rates. Participants responded both faster, *t*(23) = 2.38, *p* = 0.026, *d* = 0.49, and committed fewer errors, *t*(23) = 2.89, *p* = 0.008, *d* = 0.59, on R–E compatible (RT: *M* = 513 ms, *SD* = 60.6 ms; error rate: *M* = 4.5%, SD = 3.6%) as compared to R–E incompatible trials (RT: *M* = 536 ms, SD = 58.9 ms; error rate: *M* = 6.4%, SD = 5.0%; see Fig. 2A).

#### Anticipatory saccades

Saccades were detected according to a combined velocity (30°/s), motion (0.1°), and acceleration (8000°/${\mathrm{s}}^{2}$) threshold. Only saccades from trials with correct manual responses were considered and saccades were only assessed during the anticipatory interval between target offset and effect onset. Furthermore, saccades had to fulfill two criteria to be included in the analyses. They had to extend at least 1.0° in horizontal direction (i.e., the maximum inaccuracy accepted during tracking validation; 1,630 saccades, 14.6%, were excluded due to this amplitude criterion). Furthermore, only saccades of trials in which the first saccade during the anticipatory interval started at the screen center (± 1.0° horizontally; i.e., the position of the target; 673 saccades, 6.0%, were excluded) were included in the analyses to ensure that participants had perceived the targets and had been able to plan their responses accordingly. 8827 remaining saccades (79.3%) were included in our analyses. One participant did not perform any saccades that fulfilled the inclusion criteria during the anticipatory interval and was therefore excluded from all saccade analyses. Participants were included in the respective saccade analysis as long as they had performed at least 2 saccades per condition that fullfilled all inclusion criteria for the respective analysis (minimum saccades per condition: 4; in the R–E incompatible condition, 2 participants performed < 10 saccades that fulfilled the inclusion criteria).

On average, participants performed 384 saccades (SD = 221.1) that fulfilled all inclusion criteria. When counting only participants’ first/last effect-congruent saccade per trial (analyses of saccade amplitude, saccade-effect position difference, and saccade latency), participants, on average, performed 285 saccades (SD = 135.5) that fufilled all inclusion criteria.

#### Relative saccade frequency

**Fig. 3:**
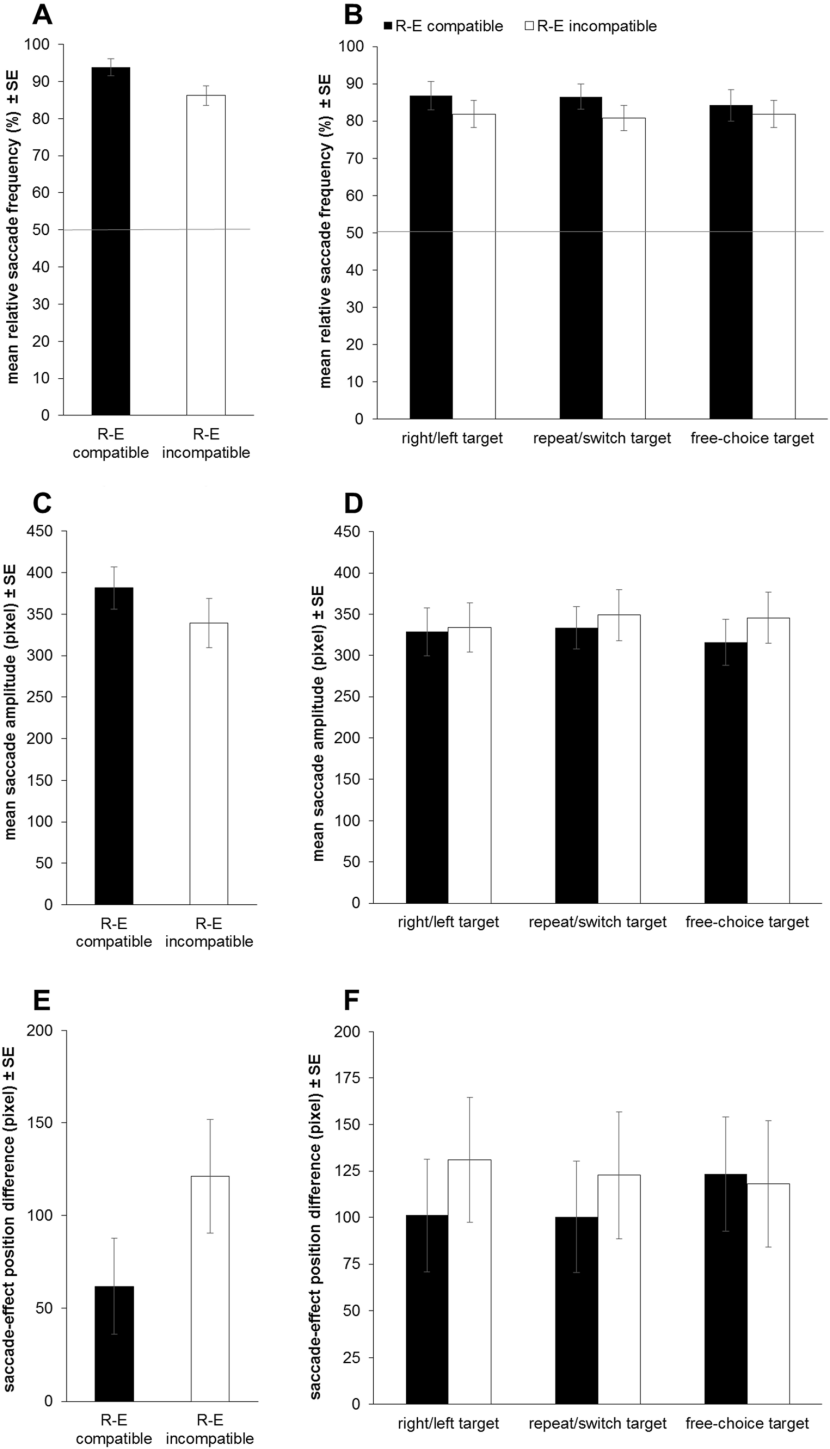
Overview over relative saccade frequencies (i.e., the size of the saccade-effect-congruency, SEC, effect, individual relative saccade frequency/ SEC score = $\frac{{N}_{\mathrm{effect}-\mathrm{congruent }}}{{N}_{\mathrm{effect}-\mathrm{congruent }}+ {N}_{\mathrm{effect}-\mathrm{incongruent }}} \mathrm{x }100\mathrm{ \%}$; **A**, **B**), mean saccade amplitudes (**C**, **D**), and mean saccade-effect position difference (**E**, **F**) in Experiments 1 (**A**, **C**, **E**) and 2 (**B**, **D**, **F**). The results of Experiment 1 are displayed separately for the two R–E compatibility conditions (R–E compatible vs. R–E incompatible) and the results of Experiment 2 are displayed separately for the two R–E compatibility conditions and the three target types (forced choice right/left vs. forced choice repeat/switch vs. free choice). Error bars indicate standard errors of the mean

A one-sample t test then determined that participants mean SEC score (*M* = 91.3%, SD = 9.7%) was significantly (and considerably) larger than 50%, *t*(22) = 20.53, *p* < 0.001, *d* = 4.28, the value expected if participants' eye movements had occurred at random. All participants showed SEC scores above 50% (range 58.8% to 100.0%). Subsequently, a paired t test was used to compare participants' SEC scores on R–E compatible and R–E incompatible trials. SEC scores were on average significantly larger on R–E compatible (*M* = 93.8%, SD = 11.0%) than on R–E incompatible trials (*M* = 86.2%, SD = 12.7%), *t*(22) = 3.00, *p* = 0.007, *d* = 0.62 (see Fig. 3A).

Importantly, action selection difficulty differs between forced choice repeat and switch targets. To rule out that the different targets (i.e., differences in action selection difficulty) yielded an essential influence on SEC scores, we post hoc conducted a 2 × 2 repeated-measures analysis of variance (ANOVA) with the within-subject factors R–E compatibility (R–E compatible vs. R–E incompatible) and target (repeat vs. switch). Confirming the previous paired t test, this ANOVA found a significant effect of R–E compatibility, *F*(1, 22) = 8.11, *p* = 0.009, ${{\eta }_{p}}^{2}$ = 0.27. The main effect of target and the interaction between R–E compatibility and target failed to reach significance, *F*s < 1, ${\mathrm{BFs}}_{01}$ ≥ 3.28. Note that the number of saccades per condition also did not significantly differ between repeat (*M* = 193, SD = 112.0) and switch targets (*M* = 191, SD = 111.1), *t*(22) = 0.34, *p* = 0.739, *d* = 0.07, ${\mathrm{BF}}_{01}$ = 4.49, in a post hoc paired t test.

Overall SEC scores larger than 50% can be interpreted as evidence in favor of anticipatory saccades towards the future position of the effect (i.e., more effect-congruent saccades than effect-incongruent saccades). However, a comparison of SEC scores between conditions should be interpreted with caution, as multiple (different) saccade measures (frequency of occurrence, latency, amplitude, and spatial accuracy) could indicate better/worse or more/less efficient proactive effect monitoring on a trial. For instance, a pattern similar to a speed–accuracy tradeoff, sometimes observed between manual RTs and error rates, could emerge. To ensure that larger SEC scores really indicated more efficient proactive effect monitoring, we therefore additionally analyzed the mean amplitude of participants' first effect-congruent saccade on a trial, the mean distance between participants' last effect-congruent saccade on a trial and the effect, and the mean latency of participants' first effect-congruent saccade. If the pattern of results is in favor of more efficient proactive effect monitoring on all measures (i.e., larger SEC scores, larger mean amplitudes, smaller position differences, and shorter saccade latencies) or there is at least evidence for more efficient proactive effect monitoring on some measures and no contrary evidence on the others, we can assume that proactive effect monitoring was indeed more efficient in one condition than the other.

#### Saccade amplitude and saccade-effect position difference

 Paired t tests comparing R–E compatible and R–E incompatible trials were additionally conducted on the mean amplitude of participants' first effect-congruent saccade on each trial and the mean position difference between participants' last effect-congruent saccade endpoint on a trial and the position of the effect (effect center) on that trial. Mean saccade amplitudes were significantly larger for R–E compatible as compared to R–E incompatible trials, *t*(22) = 2.66, *p* = 0.014, *d* = 0.55 (see Fig. 3C). Furthermore, participants' last effect-congruent saccades landed significantly closer to the effect on R–E compatible than on R–E incompatible trials, *t*(22) = 3.27, *p* = 0.004, *d* = 0.68 (see Fig. 3E).

#### Saccade latency

**Fig. 4:**
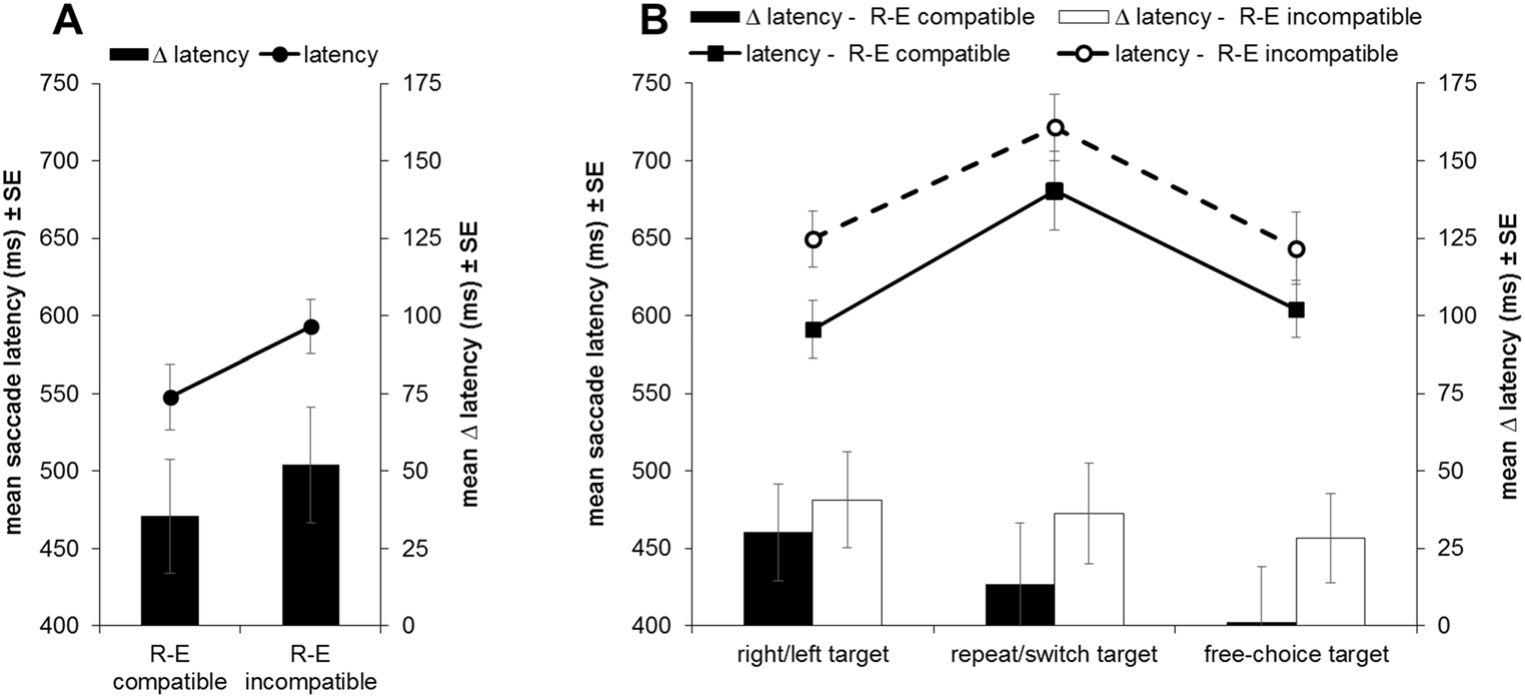
Mean latency of the first effect-congruent saccades in each trial as well as saccade-manual latency differences (∆$\mathrm{latency}$ = ${\mathrm{latency}}_{\mathrm{saccade}}$−${\mathrm{latency}}_{\mathrm{manual}}$) **A** in Experiment 1 displayed separately for the two R–E compatibility conditions (R–E compatible vs. R–E incompatible) and **B** in Experiment 2 displayed separately for the two R–E compatibility conditions and three target types (forced choice right/left vs. forced choice repeat/switch vs. free choice). Error bars indicate standard errors of the mean

 A paired t test showed that participants' first effect-congruent saccade on each trial on average occurred earlier on R–E compatible as compared to R–E incompatible trials, *t*(22) = 2.84, *p* = 0.010, *d* = 0.59 (see Fig. 4A).

It is important to note that although manual responses and anticipatory saccades reflect different processes—action selection and proactive effect monitoring, respectively—their latencies show substantial within-subject correlations (Experiment 1: mean *r* = 0.70—back-transformed from Fisher-transformed values; one-sample t test of the Fisher-transformed within-subject correlations against 0: *t*(22) = 13.61, *p* < 0.001, *d* = 2.84; see also Pfeuffer et al., 2016). This suggests that both processes are temporally coordinated to a certain extent (Pfeuffer et al., 2016). For instance, one might presume that anticipatory saccades are delayed until a certain threshold of manual response preparation has been exceeded. Saccade latencies might therefore in parts reflect effects observed in manual response latencies. It is thus essential to not only assess the latency of participants’ first effect-congruent saccade per trial, but to also examine the latency of participants’ first effect-congruent saccade per trial relative to the manual RT on the respective trial.

To account for potential influences of manual actions on the latency of anticipatory saccades and partial them out, we therefore additionally conducted a paired *t* test on participants' saccade-manual latency differences (∆ latency = ${\mathrm{latency}}_{1\mathrm{st effect}-\mathrm{congruent\,\, saccade}}-{\mathrm{latency}}_{\mathrm{manual \,\,response}}$). Saccade-manual latency differences did not significantly differ between R–E compatible and R–E incompatible trials, *t*(22) = 1.31, *p* = 0.203, *d* = 0.27, ${\mathrm{BF}}_{01}$ = 2.14 (two-sided; see Fig. 4A).

### Discussion

Experiment 1 investigated whether anticipatory saccades could also be observed in a forced choice setting in which targets did not predict upcoming effects. Instead of using targets that directly indicate the required response, we introduced repeat/switch targets that indicated whether participants had to perform the same response as on the previous trial or the opposite response. Apart from this manipulation, Experiment 1 was comparable to the forced choice experiments conducted in Pfeuffer et al. (2016).

In line with previous studies on spatial R–E compatibility (e.g., Kunde, 2001), participants were faster to respond (manually) in R–E compatible as compared to R–E incompatible conditions. This suggests that (as also indicated by the anticipatory saccades) they anticipated the effects of their actions. This effect anticipation influenced manual action selection.

Most importantly, we found a large SEC effect. This shows that our participants performed many more saccades in the direction in which the future effects of their actions would appear than in the opposite direction. Thus, we replicated the central finding of Pfeuffer et al. (2016). Our participants clearly anticipated the effects their actions would have and proactively monitored them by performing anticipatory saccades. This further indicates that anticipatory saccades in the forced choice experiments of Pfeuffer et al. (2016) cannot be attributed to target–effect associations alone. In contrast, anticipatory saccades towards future effects of own actions are, to a substantial extent, performed based on action–effect associations and the corresponding anticipations. In Experiment 2, we assessed whether target–effect associations nevertheless played a role in addition.

## Experiment 2

Building on the findings of Experiment 1, Experiment 2 addressed the question whether anticipatory processes (reflected in spontaneous, uninstructed, anticipatory saccades towards future effects) differed between forced choice targets that directly allowed for versus did not directly allow for effect predictions. That is, we assessed whether anticipatory saccades differed between conditions in which target–effect associations could (forced choice right/left targets like in Pfeuffer et al., 2016) versus could not (forced choice repeat/switch targets) play a role.^4^

Importantly, in research on the effects of R–E compatibility on manual action selection processes, there has been a debate on whether there are differences between a stimulus-based (forced choice) and an intention-based (free choice) action mode (see e.g., Herwig et al., 2007; Herwig & Waszak, 2012; Pfister et al., 2010, 2011; Waszak et al., 2005). Recent findings suggested that action–effect associations and effect anticipation (e.g., Janczyk et al., 2012, 2017; Pfister & Kunde, 2013) as well as (reactive) effect monitoring (Wirth et al., 2018) do not differ between action modes. However, more direct evidence for this claim as well as information on similarities/differences between action modes in terms of proactive effect monitoring are missing. Based on the prior findings of Wirth et al. regarding reactive effect monitoring, we hypothesized that forced choice and free choice action modes also do not differ regarding concurrent, proactive monitoring processes originating from effect anticipations. Thus, we compared the two forced choice conditions (effect-unpredictive and effect-predictive targets) to a free choice condition (effect-unpredictive targets) to assess the influence of action mode on anticipatory saccades (i.e., proactive effect monitoring processes) in goal-directed action control. Given that we hypothesized not to find a difference, we additionally conducted Bayesian analyses.

### Methods

#### Participants

 We estimated the effect size of possible differences between forced choice and free choice action modes on the basis of the SEC effects observed in Pfeuffer et al. (2016) for forced choice (*η*_*p*_^*2*^ = 0.50) and free choice actions (*η*_*p*_^*2*^ = 0.89, ∆ *η*_*p*_^*2*^ = 0.39). Thus, we expected an effect size of *η*_*p*_^*2*^ = 0.39 for the crucial main effect of target type on participants’ SEC scores in the planned 2 × 3 (R–E compatibility x target type) repeated-measures ANOVA on SEC scores. Our a priori sample size estimation (GPower, Erdfelder et al., 1996; Faul et al., 2007) indicated that 16 participants were sufficient to find an effect of *η*_*p*_^*2*^ = 0.39 with α = 0.05 and a power of 80%. As there were no prior data giving an indication of the size of potential contributions of target–effect associations, we decided to collect a sample of 24 participants as in Experiment 1.

Twenty-four new participants (20 female, 4 male, 1 left handed, 3 left eye dominant, mean age = 22.1, SD = 3.1) who provided written informed consent and took part in the experiment either for course credit or a financial compensation of 10€ were included in our analyses. All participants included in our analyses had normal or corrected-to-normal vision. Two additional participants were replaced due to issues with tracking their eyes leading to partial signal loss.

#### Stimuli and apparatus

 The setting of Experiment 2 was equivalent to Experiment 1.

#### Design and procedure

 The design and procedure of Experiment 2 were equivalent to Experiment 1 with two exceptions (see Fig. 1B for the trial structure of Experiment 2). First, Experiment 2 consisted of 12 blocks of 60 trials and a preceding practice block of 60 trials. Second, in each block, three target types (forced choice right/left vs. forced choice repeat/switch vs. free choice; 0.7°, displayed in white) appeared randomly intermixed and in equal proportion. Forced choice right/left targets were a triangle and a square that directly indicated whether participants were to press a right or left response. Forced choice repeat/switch targets were equivalent to Experiment 1 (“ = ” vs. “x”; right/left pointing arrow on the first trial of each block). Free choice trials were indicated by a “#”. Participants were instructed to spontaneously choose their response on these trials without following a distinct pattern. They were told to imagine flipping a coin every time they saw a free choice target and to try and achieve about an equal number of right and left responses overall. At the end of each block, they were additionally informed about the number of left/right free choice keypresses they had made.

### Results

#### Data preparation and analyses

Again, the first trial of each block as well as premature (< 0.1%) and omitted responses (0.1–0.5% across target types) were excluded from all analyses. Trials with errors (forced choice right/left targets: 3.6%; forced choice repeat/switch targets: 9.2%) were excluded from RT and saccade analyses.

Importantly, in Experiment 2, we examined whether there were no differences between target types regarding anticipatory saccade measures. To gain evidence in favor of these hypothesized null effects for the main effect of target type and the interaction of R–E compatibility and target type, we therefore additionally conducted Bayesian Repeated-Measures ANOVAs with default prior scales using JASP (version 0.8.0.0, Love et al., 2015; see Rouder et al., 2009, 2017, for information on Bayesian statistics) for all non-significant effects.

#### Manual responses

 On free choice trials, the frequency of right (*M* = 50.1%, *SD* = 1.9%) and left (*M* = 49.9%, SD = 1.9%) response choices did not significantly differ, *t*(23) = 0.16, *p* = 0.870, *d* = 0.03.

RT outliers (i.e., trials deviating by more than 3 SDs from their individual cell means; 1.3%) were excluded from RT analysis. A 2 × 3 repeated-measures ANOVA with the within-subject factors R–E compatibility (R–E compatible vs. R–E incompatible) and target type (forced choice right/left target vs. forced choice repeat/switch target vs. free choice target) was conducted on RTs and a 2 × 2 repeated-measures ANOVA with the factors R–E compatibility (R–E compatible vs. R–E incompatible) and target type (forced choice right/left target vs. forced choice repetat/switch target) was conducted on error rates (see Fig. 2B for the results).

For RTs, the main effect of R–E compatibility, *F*(1,23) = 15.75, *p* = 0.001, ${{\eta }_{p}}^{2}$= 0.41, was significant with faster responses on R–E compatible as compared to R–E incompatible trials. The main effect of target type also reached significance, *F*(2,46) = 35.49, *p* < 0.001, ${{\eta }_{p}}^{2}$= 0.61. Contrasts showed that RTs for forced choice repeat/switch targets were significantly larger than for forced choice right/left targets, *F*(1,23) = 50.77, *p* < 0.001, ${{\eta }_{p}}^{2}$= 0.69. Furthermore, RTs for forced choice repeat/switch targets were also significantly larger than RTs for free choice targets, *F*(1,23) = 88.84, *p* < 0.001, ${{\eta }_{p}}^{2}$= 0.79. The interaction of R–E compatibility and target type was significant, *F*(2,46) = 3.63, *p* = 0.034, ${{\eta }_{p}}^{2}$= 0.14. Paired t tests examined R–E compatibility effects separately for the three target types. They showed that responses were significantly faster on R–E compatible than R–E incompatible trials for forced choice right/left targets, *t*(23) = 4.79, *p* < 0.001, *d* = 0.98, and forced choice repeat/switch targets, *t*(23) = 3.93, *p* = 0.001, *d* = 0.80, but not for free choice targets, *t*(23) = 1.82, *p* = 0.082, *d* = 0.37, ${\mathrm{BF}}_{01}$ = 1.13 (two-sided), were used.

In error rates, the main effect of R–E compatibility showed a non-significant trend towards larger error rates on R–E incompatible as compared to R–E compatible trials, *F*(1,23) = 3.49, *p* = 0.075, ${{\eta }_{p}}^{2}$= 0.13, ${\mathrm{BF}}_{01}$ = 2.16. Again, the main effect of target type was significant, *F*(1,23) = 26.09, *p* < 0.001, ${{\eta }_{p}}^{2}$= 0.53, with participants committing significantly more errors for forced choice repeat/switch targets than forced choice right/left targets. The interaction of R–E compatibility and target type did not approach significance, *F* < 1, ${\mathrm{BF}}_{01}$ = 3.52.

#### Anticipatory saccades

 Saccades were detected and treated as in Experiment 1. 3,333 saccades (20.9%) were excluded as they did not meet the amplitude criterion and 536 saccades (3.3%) were excluded as the first saccade after target offset did not start at (or near) the screen center. 12,072 saccades (75.7%) were included in the analyses. Participants were included in the respective saccade analysis as long as they had performed at least 2 saccades per condition that fullfilled all inclusion criteria for the respective analysis (minimum saccades per condition: 2; across all 24 participants × 2 R–E compatibility conditions × 3 target type conditions, a total of 10 cells that belonged to 3 participants contained < 10 saccades that fulfilled the inclusion criteria).

On average, participants performed 503 saccades (SD = 307.3) that fulfilled all inclusion criteria. When counting only participants’ first/last effect-congruent saccade per trial (analyses of saccade amplitude, saccade-effect position difference, and saccade latency), participants, on average, performed 396 saccades (SD = 194.6) that fulfilled all inclusion criteria.

#### Relative saccade frequency, saccade amplitudes, and saccade-manual position differences

 A one-sample t test determined that participants' overall SEC scores (*M* = 84.6%, *SD* = 13.9%) were significantly above chance level, *t*(23) = 12.16, *p* < 0.001, *d* = 2.48. All participants showed SEC scores above 50% (range 52.7% to 98.7%).

2 × 3 Repeated-measures ANOVA compared SEC scores, mean amplitudes, and mean position differences between the levels of the within-subject factors R–E compatibility (R–E compatible vs. R–E incompatible) and target type (forced choice right/left vs. forced choice repeat/switch vs. free choice). Note that only 22 participants provided sufficient data in each condition (at least 2 saccades per condition) to be included in the analyses of mean amplitudes and mean position differences.

For SEC scores, neither main effect nor the interaction reached significance, *F*s ≤ 1.65, *p*s ≥ 0.212, ${{\eta }_{p}}^{2}$ =0.07, target type: ${\mathrm{BF}}_{01}$ = 13.39, R–E compatibility X target type: ${\mathrm{BF}}_{01}$ = 8.32 (see Fig. 3B). Similarly, neither of the main effects nor the interaction was significant for participants' mean amplitudes, *F*s ≤ 2.08, *p*s ≥ 0.137, ${{\eta }_{p}}^{2}$ =0.09, target type: ${\mathrm{BF}}_{01}$ = 7.86, R–E compatibility X target type: ${\mathrm{BF}}_{01}$ = 4.66 (see Fig. 3D). For mean saccade-effect position differences, neither of the main effects were significant, *F*s ≤ 1.46, *p*s ≥ 0.241, ${{\eta }_{p}}^{2}$ =0.07, target type: ${\mathrm{BF}}_{01}$ = 9.70. However, the interaction between R–E compatibility and target type reached significance, *F*(1,21) = 3.68, *p* = 0.034, ${{\eta }_{p}}^{2}$= 0.15 (see Fig. 3F). Note, however, that the Bayes factor ${\mathrm{BF}}_{01}$= 1.96 still showed a small tendency towards the null hypothesis. The R–E compatibility effect was significant neither for forced choice right/left targets, *t*(21) = 1.74, *p* = 0.096, *d* = 0.37, ${\mathrm{BF}}_{01}$ = 3.04 (two-sided), nor for forced choice repeat/switch targets, *t*(21) = 1.65, *p* = 0.114, *d* = 0.35, ${\mathrm{BF}}_{01}$ = 1.38 (two-sided), nor for free choice targets, *t*(21) = -0.37, *p* = 0.713, *d* = 0.08, ${\mathrm{BF}}_{01}$ = 3.66 (two-sided).

#### Saccade latency

 Twenty-two participants provided sufficient data in all conditions to be included in the analyses of saccade latencies and saccade-manual latency differences. 2 × 3 Repeated-measures ANOVAs with the within-subject factors R–E compatibility and target type were conducted on the dependent measures.

Participants' saccade latencies were significantly prolonged in R–E incompatible as compared to R–E compatible trials, *F*(1,21) = 6.24, *p* = 0.021, ${{\eta }_{p}}^{2}$= 0.23 (see Fig. 4B). Furthermore, saccade latencies significantly differed between the target types, *F*(1,21) = 20.16, *p* < 0.001, ${{\eta }_{p}}^{2}$= 0.49, ${\mathrm{BF}}_{01}$ < 0.01. Contrasts showed that saccade latencies for forced choice repeat/switch targets were significantly larger than for forced choice right/left targets, *F*(1,21) = 30.11, *p* < 0.001, ${{\eta }_{p}}^{2}$= 59. Furthermore, saccade latencies for forced choice repeat/switch targets were also significantly larger than saccade latencies for free choice targets, *F*(1,21) = 27.35, *p* < 0.001, ${{\eta }_{p}}^{2}$= 0.57. The interaction of R–E compatibility and target type did not reach significance, *F* < 1, ${\mathrm{BF}}_{01}$ = 6.48.

Again, saccade latencies and manual RTs showed substantial within-subject correlations (mean *r* = 0.66—back-transformed from the Fisher-transformed values; one-sample t test of the Fisher-transformed within-subject correlations against 0: *t*(21) = 17.12, *p* < 0.001, *d* = 3.65; comparison between target types: *F* < 1, ${\mathrm{BF}}_{01}$= 7.11). Given that we found differences between target types in manual RTs, it was essential to additionally assess saccade-manual latency differences for a measure of saccade timing unaffected by temporal coordination with manual responses.

For saccade-manual latency differences, neither of the main effects nor the interaction was significant, *F*s ≤ 1.72, *p*s ≥ 0.204, ${{\eta }_{p}}^{2}$ =0.08, target type: ${\mathrm{BF}}_{01}$ = 4.18, R–E compatibility X target type: ${\mathrm{BF}}_{01}$ = 6.38.

### Discussion

Experiment 2 first examined whether anticipatory saccade measures indicating proactive effect monitoring processes differed between forced choice targets that did versus did not directly predict the upcoming effect. Second and most important, we compared these forced choice targets to free choice targets to assess a potential additional influence of the action mode (forced choice/stimulus-based vs. free choice/intention-based) on proactive effect monitoring. This comparison was conducted to gain further information about differences between forced choice and free choice actions to inform the debate on potential differences between these action modes.

In manual responses, we found both an R–E compatibility effect (e.g., Kunde, 2001; not significant for free choice trials) and an effect of target type. These effects were mirrored in saccade latencies, but not saccade-manual latency differences which assess saccade timing unconfounded by influences on manual responses that could have propagated to saccades.

Again, across conditions, we found an SEC effect with significantly more effect-congruent than effect-incongruent saccades overall. Replicating Pfeuffer et al. (2016) as well as Experiment 1, this finding indicates that participants anticipated the future effects of their actions and thus looked towards the future positions of these effects. In line with Pfeuffer et al. (2016), we interpret this finding as evidence for the idea that anticipatory saccades reflect proactive effect monitoring.

Importantly, neither participants' SEC scores nor mean saccade amplitudes were affected by target type, a finding supported by Bayesian evidence in favor of the null hypothesis. Regarding the distance between participants' last effect-congruent saccades and the position of the effect, we found a small interaction effect between R–E compatibility and target type. However, a Bayesian analysis of this effect still indicated evidence in favor of the null hypothesis. We thus conclude that this interaction was likely a spurious result.

Overall, our findings thus indicate that, first, in anticipatory saccades, there was no difference between forced choice targets that predicted (forced choice right/left targets) versus did not predict (forced choice repeat/switch targets) the upcoming effect. This suggests that anticipatory saccades occurring in forced choice settings are driven by action–effect associations and not influenced by target–effect associations. Even in forced choice settings using effect-predictive targets, anticipatory saccades can therefore be interpreted as measures of effect anticipation and proactive effect monitoring without any restrictions.

Second, our findings suggest that anticipatory saccades also do not differ between forced choice (i.e., stimulus-based) and free choice (i.e., intention-based) action modes. Forced choice repeat/switch targets required participants to memorize their responses. As effects were helpful in this respect, one might argue that in Experiment 2, conditions were equated in terms of the (perceived) relevance of the effects. This might have reduced potential differences between action modes. Conversely, however, we argue that the equivalence between conditions in terms of effect relevance allowed for a fairer assessment that accounted for actual influences of action mode rather than confounds (like, e.g., differences in perceived effect relevance) introduced, for instance, by forced/free choice task instructions. Our findings therefore provide strong support against the notion that action mode itself impacts on proactive monitoring.

Thus, for the first time, we provide direct evidence that regarding processes of proactive effect monitoring, there seems to be no difference between the two action modes. This finding is also relevant to the debate on whether forced choice/stimulus-based and free choice/intention-based action modes differ in terms of their influence on action selection processes in endogenous action control (see e.g., Herwig et al., 2007; Herwig & Waszak, 2012; Janczyk et al., 2012, 2017; Pfister et al., 2011; Pfister & Kunde, 2013; Richardson et al., 2020; Waszak et al., 2005). At present, a growing number of findings suggests that action mode does not yield a crucial influence on action selection. Adding to these findings, the present experiments clearly support the notion that action mode does not influence proactive effect monitoring.

## General discussion

In two experiments, we assessed proactive effect monitoring as evidenced by anticipatory saccades towards the locations at which participants' actions would subsequently cause visual effects. Participants' left/right responses were predictably followed by visual effects (coloured circles) on the left/right that appeared after a brief R–E interval. We asked whether target–effect associations and/or action mode yielded an influence on proactive effect monitoring as evidenced by anticipatory saccades. In Experiment 1, we assessed whether anticipatory saccades could be observed when forced choice targets were themselves unpredictive of the effects (repeat/switch forced choice targets). In Experiment 2, we compared free choice targets and effect-predictive as well as effect-unpredictive forced choice targets to examine a potential influence of target type and/or action mode on proactive effect monitoring.

First, in both experiments, we found a substantial SEC effect, indicating that participants looked much more often towards the location of the future effect rather than away from it. The relative frequency of saccades towards the location of the future effect clearly exceeded the chance level (50%), suggesting that participants' effect anticipations led to overt shifts of attention towards future effect locations, that is, anticipatory saccades. These findings replicate the results of Pfeuffer et al. (2016) and further support the notion that effect anticipation can be assessed directly via spontaneous eye movements (i.e., anticipatory saccades).

Second, our results also provide clearcut answers to our main research questions. Experiment 1 demonstrated that, in a forced choice setting, participants anticipatorily looked towards their actions' future effect locations even when targets per se were unpredictive of the upcoming effects (repeat/switch targets). This indicates that anticipatory saccades occurring in forced choice settings, to a substantial degree, result from action–effect (and not target–effect) associations. Experiment 2 compared the contributions of action–effect and target–effect associations to anticipatory saccades by contrasting forced choice left/right and repeat/switch targets. Our Bayesian analyses indicated that neither SEC effects nor other saccade measures differed between these target types. As only action–effect associations can lead to anticipatory saccades for forced choice repeat/switch targets, this finding speaks against a contribution of target–effect associations to anticipatory saccades observed in goal-directed action control.

Thus, we conclude that when our actions cause predictable effects in the environment, a proactive effect monitoring process based on action–effect (and not target–effect) associations leads to attentional shifts towards future effect locations in preparation of comparing actual to expected effects. These findings provide empirical support for our previously formulated theoretical assumption (Pfeuffer et al., 2016) that anticipatory saccades originate from action–effect associations both in free choice and forced choice settings.

Most importantly, the results of Experiment 2 provided Bayesian evidence against differences between forced choice and free choice action modes in terms of proactive effect monitoring. Anticipatory saccades towards future effects in forced choice and free choice conditions did not differ. This finding also informs the debate on whether forced choice and free choice action modes differ in terms of how actions are controlled (see, e.g., Herwig & Horstmann, 2011; Herwig et al., 2007; Herwig & Waszak, 2009, 2012; Janczyk et al., 2012, 2017; Pfister et al., 2011; Pfister & Kunde, 2013; Richardson et al., 2020; Waszak et al., 2005) and monitored (Wirth et al., 2018) on the basis of anticipated effects. Recent studies indicated that action–effects were anticipated even under forced choice conditions (e.g., Janczyk et al., 2012, 2017; Pfister & Kunde, 2013; Pfister et al., 2011), a basis for effect monitoring. These studies also convincingly argued against differences between forced choice and free choice actions in terms of action–effect learning and action selection processes. Wirth et al.'s (2018) results then suggested that reactive effect monitoring might not differ between forced choice and free choice action modes, but this conclusion was drawn based on rather indirect indicators of monitoring processes. In line with these suggestions, here, we directly demonstrate that the effect anticipations we derive from action–effect associations and their reflection in attentional shifts towards the future effects of our actions do not differ between action modes. That is, proactive effect monitoring in forced choice and free choice conditions (i.e., in a stimulus-based vs. intention-based action mode) is comparable. Our study therefore provides converging evidence that not only action selection but also proactive effect monitoring does not differ between action modes. We thus further substantiate the claim that forced choice and free choice actions do not differ in these respects.

## Conclusion

In conclusion, our findings show that action–effect but not target–effect associations underly anticipatory saccades observed when our actions cause predictable effects in the environment. These action–effect associations and the resulting effect anticipations trigger two essential functional processes: action selection and proactive effect monitoring (see also Pfeuffer et al., 2016). Importantly, whether we intentionally choose our actions (free choice) or simply act as instructed (forced choice) does not affect how we anticipatorily shift our attention to monitor the future effects of our actions. That is, proactive effect monitoring—as reflected in anticipatory eye movements in the present paradigm—does not differ between free and forced choice action modes.

## Data Availability

The data of the reported experiments are available via the Open Science Framework: https://osf.io/2vfxc/; https://doi.org/10.17605/OSF.IO/2VFXC.

## Footnotes

1 Please note that *anticipatory* eye movements refer to eye movements that occur prior to the onset of one’s action ‘s future effect. That is, we refer to eye movements occuring during an anticipatory interval, during which the effect may already be predicted on the basis of the response one prepares or has already performed, but during which the effect has not yet appeared, as *anticipatory*. As such, we consider eye movements occurring prior to effect onset (i.e., during an anticipatory interval between target offset and effect onset) as based on effect anticipation even if they occur during a blank screen action–effect interval after effect-generating responses which predict the future effect with high/perfect certainty. Note that prior studies using the response–effect compatibility paradigm introduced by Kunde (2001) very often did not use delays between action and effect. As such, in these studies, the term *anticipatory* mostly referred to processes occurring *prior to the action* (which equated *prior to the effect* there). Moreover, as reaction times were assessed, any observed effects would have been due to anticipatory processes *prior to the action* in these studies. As the present study introduced delays between action and effect and assessed eye movements until effect onset, our use of the term *anticipatory* slightly differs from its common use in several prior response–effect compatibility studies. That is, we refer to anticipatory saccades whenever a saccade is performed *prior to the effect* (instead of restricting the interval to the time *prior to the action*).

2 Please note that when speaking of goal-directed action control, we refer to the manual responses described in this context that are performed to produce certain effects. The anticipatory saccades we discuss emerged without any instruction to perform a certain eye movement and we do not mean to imply that these anticipatory saccades were necessarily performed intentionally. In eye tracking research, instructed saccades in response to visual target stimuli are termed goal-directed. According to this terminology for reactive saccades, the anticipatory saccades discussed in this paper cannot be considered as “goal-directed”.

3 Please note that errors might have compromised participants’ performance not only in the erroneous trial itself but also in the subsequent trial. This could especially have affected performance in case a forced choice repeat/switch target appeared following an erroneous trial, as participants might have gotten confused about which response (correct vs. erroneous/actual response) to use as the reference in the following trial. We therefore re-computed all analyses of Experiments 1 and 2 and additionally excluded trials following a trial with an erroneous response. There were no qualitative differences between the analyses including and excluding trials following an erroneous trial, and we therefore only report the analyses including trials following an erroneous response.

4 Please note that performance differences between forced choice repeat/switch targets and forced choice right/left targets were observed (longer RTs and higher error rates for repeat/switch targets). This suggests that some processes, especially concerning manual action selection, differ between these two types of forced choice targets. Nevertheless, the essential aspect for saccade analyses within the scope of this study, namely that both target types require forced choice stimulus-based responses, is the same for the two types of forced choice targets. Their essential difference is that right/left targets themselves are predictive of the effect, whereas repeat/switch targets are not.

## Code availability

Experiment files and syntaxes are available via the Open Science Framework: https://osf.io/2vfxc/; https://doi.org/10.17605/OSF.IO/2VFXC.
